# Primary malignant melanoma of prostate

**DOI:** 10.4103/0974-7796.65105

**Published:** 2010

**Authors:** M. Doublali, A. Chouaib, A. Khallouk, M. F. Tazi, M. J. El Fassi, My. H. Farih, H. Elfatmi, M. Bendahou, A. Benlemlih, O. Lamarti

**Affiliations:** Department of Urology, University Hospital Center Hassan II, Fez, Morocco; 1 Histopathology, University Hospital Center Hassan II Fez – Morocco

**Keywords:** Melanoma, prostate, primary

## Abstract

Primary genitourinary melanoma accounts for less than one per cent of all cases of melanoma. Most cases attributed to the prostate actually originate from the prostatic urethra. Due to its infrequency, primary malignant melanoma of the genitourinary tract presents a difficult diagnostic and management challenge. We report a case of primary malignant melanoma of the prostate found during transurethral resection of the prostate.

## INTRODUCTION

Melanoma of prostatic origin is very rare. It presents a difficult diagnostic, management challenge and a very poor prognosis. We report a case of primary malignant melanoma of the prostate found incidentally following transurethral resection of the prostate.

## CASE REPORT

**Figure 1:**
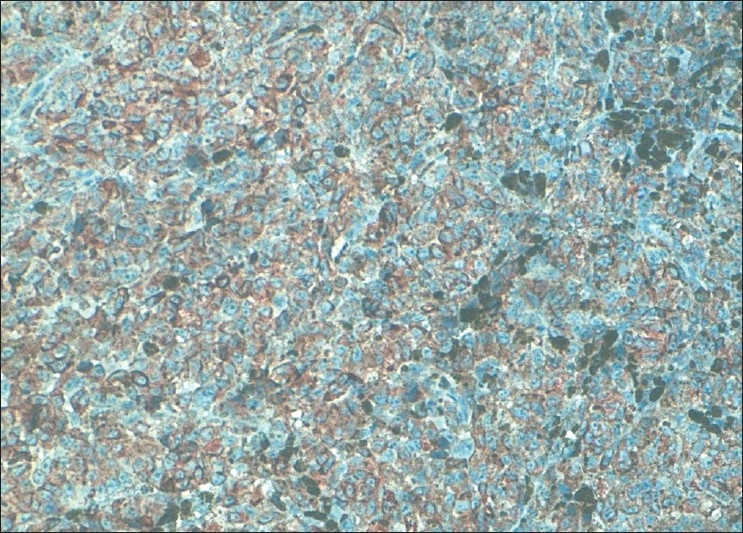
Proliferation of big tumoral cells with very atypical cores. The tumoral cells express HMB45

A 75-year-old White man was referred to the urology department for management of urinary tract obstruction. He reported an 18-month history of recurrent urinary tract infection. On examination, the patient was emaciated with stable vital signs. Digital examination and serum prostate specific antigen revealed no evidence of malignancy; urinary tract obstruction was managed with transurethral resection of the prostate. Urethroscopy had revealed a black discoloration of the prostate. Histology of the surgical specimen demonstrated melanoma cells distributed in the stroma, independent of the acinar glands with intact urethral epithelium [Figure 1]. No other focus of malignant melanoma was found after physical examination of body skin surface, mucosa, tomography of brain, abdomen and pelvic cavity, and endoscopy of gastrointestinal tract.

Radical prostatectomy with chemotherapy was suggested. However, the patient refused further surgical and systemic treatment. He was discharged and died at home a month later.

## DISCUSSION

Although the genitourinary tract is a common site of metastatic melanoma, the primary malignant melanoma of genitourinary tract comprises less than one per cent of all malignant melanomas.[1] The prostate is rarely the site of a primary malignant melanoma and the most reported cases attributable to the prostate actually have been of prostatic urethral origin or due to a metastatic lesion.[2,3] In our case, urethral epithelium was intact and the tumor was confined to the prostate. Malignant melanoma cells were distributed in prostatic stroma with normal acinar glands. Pigmentation is infrequent in surgical specimens of the prostate determined histopathologically, the major differential diagnoses are blue nevus and melanosis of the prostate. Melanosis of the prostate gland is defined by the presence of melanin containing cells in the stroma, as seen in blue nevus, plus melanin in prostatic glandular epithelial cells.[4]

In two reported cases of melanosis of the prostate, melanin was found in tumor cells of well-differentiated adenocarcinoma. We also saw a case of melanosis in which tumor cells of prostatic adenocarcinoma as well as benign epithelial cells and stromal melanocytes contained melanin.[5] Although radical surgery with or without chemotherapy has been the standard management for genitourinary tract malignant melanoma,[1] even if the prognosis is dismal, the strategy for primary malignant melanoma of the prostate cannot be determined due to rarity of this case treatment. We found that transurethral resection was not sufficient. However, our patient refused further surgical or systemic treatment.

## CONCLUSION

Melanoma of prostatic origin is extremely rare. These patients are difficult to diagnose and carry a very poor prognosis. Aggressive surgical resection is the current treatment standard.
